# Modelling digestive hydrolysis of nutrients in fish using factorial designs and desirability function

**DOI:** 10.1371/journal.pone.0206556

**Published:** 2018-11-01

**Authors:** Neda Gilannejad, Gonzalo Martínez-Rodríguez, Manuel Yúfera, Francisco J. Moyano

**Affiliations:** 1 Instituto de Ciencias Marinas de Andalucía (ICMAN-CSIC), Puerto Real, Spain; 2 Departamento de Biología y Geología, Facultad de Ciencias, Campus de Excelencia Internacional del Mar (CEI-MAR), Universidad de Almería, La Cañada de San Urbano, Almería, Spain; National Cheng Kung University, TAIWAN

## Abstract

Models simulating the *in vitro* digestive hydrolysis of nutrients by different animal species are frequently used to obtain a better understanding of factors affecting this process. Optimization algorithm of a model may be used to prospect the more favourable combination of selected factors resulting in the higher performance. This study was conducted to determine the combination of factors (pH, enzyme:substrate ratio, and reaction time) leading to highest bioavailability of proteins and carbohydrates in the gilthead seabream gastrointestinal tract. Besides, a novel multi-objective algorithm, desirability function, was introduced for optimization of the digestive hydrolysis of nutrients within the simulated gut of the species, using models based on the Response Surface Methodology. Design of experiment was defined based on the physiology and culture conditions of the species, and *in vitro* assays were performed in a two-phase (stomach ad intestine) digestion process, using the species-specific enzyme extract. According to results, intestinal phase of digestion makes the major contribution to the total protein hydrolysis, being the efficiency of the process directly correlated to all the three studied factors. In contrast, the efficiency of carbohydrate hydrolysis was directly correlated to the amount of substrate and inversely to the pH, while reaction time did not exert a significant effect. The physiological range of the factors studied in the assays favoured the hydrolysis of proteins over carbohydrates, a similar scenario to that observed in the live fish. Results from the mathematical models and their simultaneous optimization obtained from this work may have practical applications in design of feeds for this species.

## Introduction

Considering the digestive system of a vertebrate species as a complex bioreactor may provide an insight into the relative influence of different factors affecting its functionality and may help to explain the results obtained from the food hydrolysis. This information may also give orientations to improve the efficiency of the digestion process and, hence, to increase the potential bioavailability of the main nutrients. Modelling the different steps involved in digestion can be achieved from either a theoretical or a practical perspective. Penry and Jumars (1986, 1987) initiated a theoretical approach of digestion, arguing that modelling the digestive process is analogous to modelling the behaviour of chemical reactors [1, 2]. The question faced by these digestive physiologists was to discover how various gut morphologies and digestive reactions might maximize the animal’s rate of energy and nutrient gain, given a distribution of food, chemical compositions, and animal energetic costs, as boundary conditions. This challenge is very similar to the task of chemical engineers who have to evaluate the performance of reactors with different designs (gut functional morphologies), with the goal of maximizing the yield or yield rates (energy or nutrient assimilation). This theory of chemical reactors has been used by several authors to analyse the existing relations between diet composition, food processing, and gut morphology and has served to obtain interesting conclusions on how the digestion process is adapted to food nutrient availability in different animals [3, 4], including fish and other aquatic species [5, 6].

But, digestion is also a complex combination of different biochemical reactions (digestive hydrolysis and nutrient uptake), which take place simultaneously and are affected by a great number of factors that are continuously optimized by the living organism to achieve the most efficient response. From the above-mentioned theoretical perspective, optimization should try to find the value of each factor that produces the best possible response. In this context, optimization of factors using the multivariate Design of Experiment (DOE) has several advantages over the univariate procedures, taking less time, effort, and resources, and therefore facilitating large quantities of information with a minimum number of experiments [7]. DOE and the Response Surface Methodology (RSM) have been proved useful for developing, improving, and optimizing processes [8]. RSM is a powerful mathematical tool that allows constructing models that can be used to determine which combination of factors results in an optimal response. Besides offering a large amount of information from a small number of experiments, RSM enables the assessment of the effect of interactions among the factors on the response. RSM has been extensively used in analytical applications, the industry and in bioprocesses, but only has very recently been used within the framework of biological studies [9, 10].

Applying all the aforementioned concepts within the field of fish nutrition, bioaccessibility of nutrients within the fish gut can be considered being affected by a great number of factors, many of which are directly linked to the action of the enzymes. Since the relevance and effect of each of those factors may be extremely difficult to assess using *in vivo* experiments, an alternative approach could be the simplification of the digestion process through *in vitro* assays. It must be taken into account that *in vitro* digestion models are extensively used in humans and animals with two main objectives: to predict nutritional quality of different ingredients and to understand the effects of factors influencing the efficiency of the digestion process. In the first case, the main assumption underlying the assay is that the ingredient better hydrolysed by an enzyme mixture will produce better nutritional response in the target organism, being the key requirement of these assays a reasonable degree of correlation with the *in vivo* response. In the second case, the main assumption is that the model is a simplification of the complex digestive environment of the target species, being the key requirement that the operative conditions must be strongly based on physiological parameters previously determined in live organisms. In fish nutrition, while most *in vitro* digestion models are oriented to the first objective [11–14], very few studies have been oriented to the second goal [15, 16]. Moreover, with the exception of a recent paper from Gilannejad et al. (2017) [17], none has used the above-mentioned RSM approach.

The present work, based on the use of RSM and *in vitro* assays as well, tries to go one-step beyond by simulating the digestive hydrolysis of two substrates (protein and carbohydrates) within the gut of a fish species. To accomplish this purpose, after obtaining the adequate model for each response, the Composite Desirability (CD) function was applied to discover the optimum point of the involved factors that results in optimization of both responses at the same time [18]. The final objectives of the study were: i) to understand which ones of the considered factors have a greater impact on the digestive hydrolysis of protein and carbohydrates in the target species, and ii) to determine if optimal conditions for the hydrolysis of both substrates are coincident. The selected species for the study was the gilthead seabream (*Sparus aurata*), one of the marine species with the highest production in Mediterranean countries. Although several studies have used *in vitro* approaches to assess different aspects of protein hydrolysis in this species [19–21], to date no complex mathematical modelling of its digestion has been developed.

## Materials and methods

### 1. Biological material

Enzyme extracts required for the *in vitro* assays were obtained from adult gilthead seabream individuals, purchased from a local fish farm (N = 20; total biomass 49,316 g; average mass 2,466 ± 640 g), and maintained in 5,000 L tanks with flow-through water system and 14 h light/10 h dark photoperiod, at the ICMAN experimental facilities (REGA ES110280000311). Fish were fed *ad libitum* with a single meal (commercial feed) in the morning and were sampled at two different moments (4 and 8 hours after feeding), to ensure maximum presence of digestive enzymes both in the stomach and intestine [22]. Fish were anaesthetized and then killed with 2-phenoxyethanol overdose, and were immediately dissected to separate the stomach and the pyloric caeca + proximal intestine. Manipulation of fish was performed in accordance with the Guidelines from the European Union Council (2010/63/EU) and the Spanish legislation for the use of laboratory animals, with approval of the of the Spanish National Research Council Bioethics Committee for the project EFISHDIGEST (AGL2014-52888-R).

Enzyme extracts required for the *in vitro* assays were prepared by mechanical homogenization of the tissues in distilled water (1:10 w/v) followed by centrifugation (3,220 × g, 20 min, 4°C). The supernatant was then filtered through a dialysis system with a MWCO of 10 kDa (Pellicon XL, Millipore ^®^) and the concentrated extracts were freeze-dried until being used in the assays. Pepsin activity was determined in the stomach extract following the methodology of Anson (1938) [23], using haemoglobin as substrate. In the intestinal extract, total alkaline protease activity was measured according to the Kunitz's method (1947) [24] modified by Walter (1984) [25], using casein as substrate. One unit of enzyme activity (U) was defined as the amount of enzyme needed to catalyse the formation of 1 μg of tyrosine per minute. Additionally, total amylase activity was measured at pH 7.5, following the 3,5-di-nitrosalicylic acid (DNSA) method [26], using starch as substrate. One unit of amylase activity was defined as the amount of enzyme needed to catalyse the formation of 1 μg of maltose equivalent per minute.

### 2. *In vitro* assays

*In vitro* assays involving both stomach (acid) and intestine (alkaline) phase of digestion were performed using membrane bioreactors modified from that described in Morales and Moyano (2010) [27]. The device consists of two chambers separated by a semi-permeable membrane of 3,500 kDa MWCO (ZelluTrans/Roth^®^). Enzymes and substrates are placed in the upper chamber and maintained under continuous agitation using a magnetic stirrer. Hydrolysis products passing across the membrane into the lower chamber can be recovered at different time intervals during the reaction time. The substrate used in the assays was a mixture of pure bovine haemoglobin and pure potato starch in proportions suitable to provide a similar content in protein and carbohydrates to that of a commercial feed for this species (45% and 17%, respectively). During the acid phase of digestion, the upper chamber contained the substrate dissolved in water and adjusted to pH 4.0 as well as the crude enzyme extract from the seabream stomach while the lower chamber contained distilled water. During the alkaline phase, pH of the upper chamber was raised to any of the desired values required in the experimental design prior to the addition of the intestinal enzyme extracts, being the lower chamber filled with 100 mM Tris-Maleate buffer at the same pH (supplemented with 100 mM CaCl_2_ and 50 mM NaCl). The complete arrangement was maintained at 25°C. Total amino acids and reducing sugar released during the hydrolysis were measured using the o-phtaldialdehyde [28] and DNSA [29] methods, respectively.

### 3. Experimental design and statistical analysis

**Fig 1:**
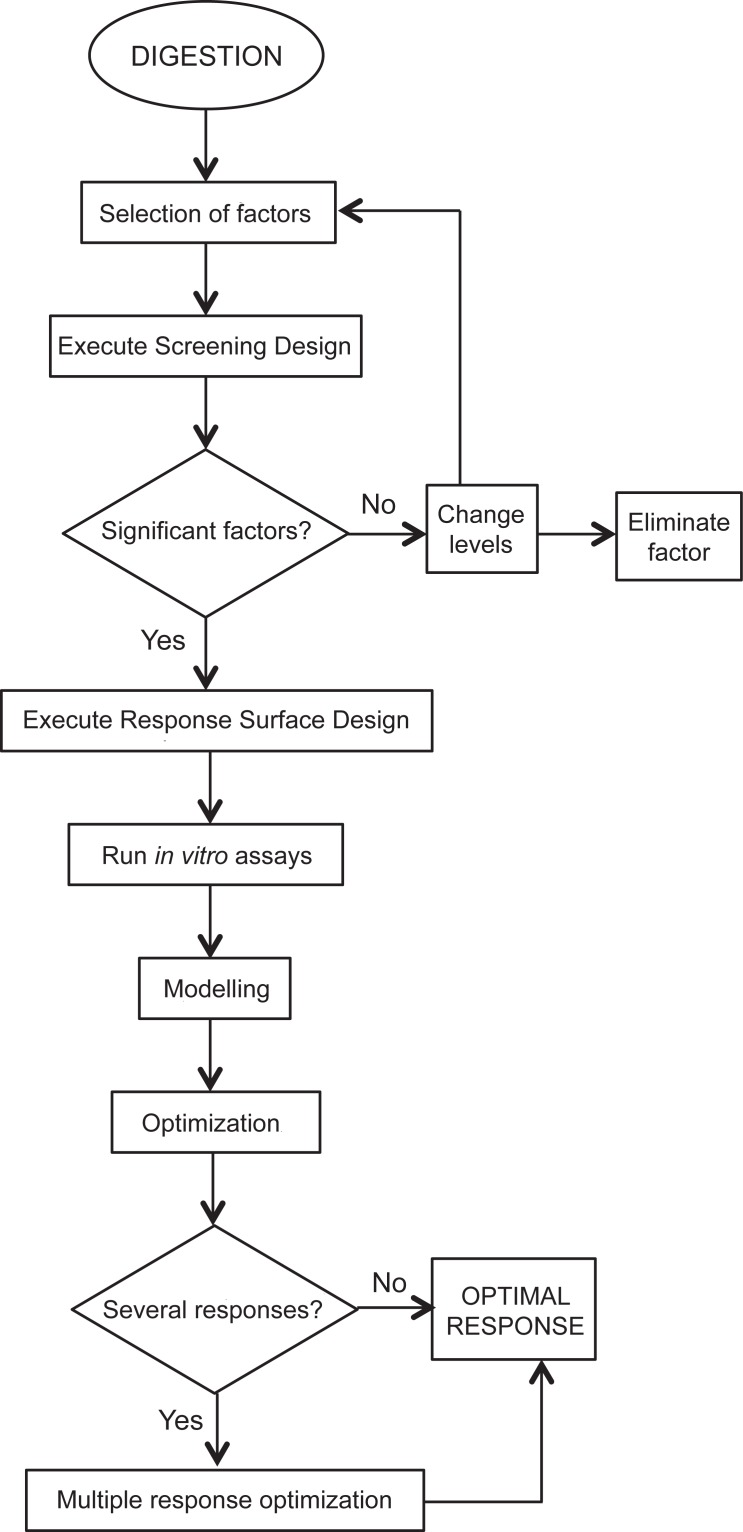
Flow chart of the steps to develop theoretical models for nutrient hydrolysis in the gilthead seabream GIT and their optimization (based on Candioti et al., 2014 [7]).

The steps followed to develop the theoretical model of protein and carbohydrate hydrolysis by the digestive enzymes of gilthead seabream are presented in the flow chart depicted in Fig 1.

#### 3.1. Selection of the factors

A preliminary assessment of the relevance of several factors, potentially affecting nutrient bioaccessibility in the fish gut, was carried out. For this purpose, three key factors (pH, reaction time and enzyme:substrate ratio) in both stomach and intestinal phases of digestion were evaluated. The justification of their selection was as follows:

*pH;* gastrointestinal pH is a relevant factor involved in several digestive processes such as enzymatic hydrolysis and solubilisation of proteins [30, 31], playing an important role in gastric fish [14]. For this reason, physiological ranges of pH both in stomach (3.5–6.5) and intestine (6.5–8.5) pH (Table 1) were established according to the published data for this species [22, 32, 33].

**Table 1:** Variables used in the experimental designs.

Variable	Range
*pH*	
Stomach	3.5–6.5
**Intestine**	**6.5–8.5**
*Enzyme*: *substrate ratio (E*:*S)*	
Stomach	125–500 U mg^−1^ protein
**Intestine**	**50–200 U mg**^**−1**^ **protein**
*Reaction time (T)*	
Stomach	4–8 h
**Intestine**	**6–8 h**

Factors used in the Central Composite Design are highlighted in bold.

*Enzyme*:*substrate ratio (E*:*S);* variable amounts of substrate may be exposed to a given amount of enzyme in the gut due to the fluctuations in the digestive enzyme production (e.g. circadian rhythms [22, 34] and the differences in the food intake (feeding protocols). In the present work, enzyme:substrate ratios were estimated for a 80 g fish, being calculated considering:

- the range of values of total protease and amylase, released during gastric and intestinal digestion in fish of that size, measured in a previous experiment,
- the amount of protein in a meal, calculated by considering the daily ration for such fish size and the mean protein content of a commercial feed (EFICO Kappa 473; Biomar Group, Denmark).

The different E:S required for the stomach (125–500 U mg^−1^ protein) and intestine (50–200 U mg^−1^ protein) were obtained after maintaining a fixed amount of substrate (290 and 110 mg of haemoglobin and starch, respectively) and changing the amount of enzyme extracts used in the assays (Table 1).

*Reaction time (T);* it was considered because gut transit rate determines the food residence time under the acid and alkaline digestion environment and, therefore, influences the hydrolysis efficiency [22]. In this study, total stomach and intestine reaction times (4–8 h and 6–8 h, respectively), were defined according to the available data on the food permanence in juvenile *S*. *aurata* (150 g) gastro intestinal tract (GIT) [33] and common feeding frequencies used in the farms for growing fish (Table 1).

A Plackett-Burman factorial design (6 factors, 1 replicate, 1 block, 14 total runs) was used for this preliminary screening (Table 2). Due to the major importance of protein digestion in fish nutrition, only hydrolysis of this substrate was considered as the response variable and the criteria for the selection of the significant factors considered for developing the model, using RSM in the next step.

**Table 2:** The preliminary Plackett-Burman Design and the values obtained for the response variable (AA).

Run order	Stom. pH	Int. pH	Stom. E:S (U mg^−1^ protein)	Int. E:S (U mg^−1^ protein)	Stom. T (h)	Int. T (h)	AA (mg)	% AA	RS (mg)	% RS
1	6.5	8.5	500	50	4	6	135	47	11	10
2	3.5	6.5	500	200	8	6	152	52	13	11
3	5	7.5	313	125	6	7	167	58	18	16
4	6.5	6.5	125	50	8	6	101	35	21	19
5	6.5	6.5	500	50	4	8	136	47	14	13
6	3.5	8.5	500	200	4	6	167	58	13	11
7	3.5	8.5	500	50	8	8	172	59	16	14
8	6.5	6.5	500	200	8	8	142	49	13	12
9	3.5	8.5	125	50	8	8	164	57	14	13
10	6.5	8.5	125	200	4	8	212	73	15	14
11	5	7.5	313	125	6	7	174	60	16	15
12	3.5	6.5	125	200	4	8	153	53	15	13
13	3.5	6.5	125	50	4	6	127	44	18	16
14	6.5	8.5	125	200	8	6	161	56	17	16

#### 3.2. Construction of the models

Further evaluation of the influence of the selected significant variables was carried out to construct a predictive model for protein and carbohydrate digestibility using RSM. For this purpose, factors that had shown negligible effects were maintained at a constant average value (T = 4 h, pH = 5, and E:S = 254 U mg^-1^ protein), while the rest of the factors were considered as independent variables. Two response variables (release of amino acids, AA, and reducing sugars, RS) were evaluated using a Central Composite Design (see Results - 2. Construction of the predictive models). Empirical equations, describing the relation between the release of amino acids or reducing sugars and the aforementioned parameters were developed. The general form of the polynomial equations (Eq 1) is:

$$Y=\beta_{0}{+\beta }_{1}X_{1}+\beta_{2}X_{2}{+\beta }_{3}X_{3}{+\beta }_{12}X_{1}X_{2}{+\beta }_{13}X_{1}X_{3}{+\beta }_{23}X_{2}X_{3}+\beta_{11}X_{1}^{2}{+\beta }_{22}X_{2}^{2}{+\beta }_{33}X_{3}^{2}$$ (1)

where Y is the response variable (either mg of AA or RS), X1, X2, and X3 are the independent variables and β0, β1, β2, β3, β11, β22, β33, β12, β13, and β23 are the regression coefficients for intercept, linear, quadratic, and interaction terms, respectively. Regression coefficients were tested using the ANOVA. After a first evaluation, models were simplified by removal of any non-significant term (*p* > 0.05). Surface plots showing the combined effect of each pair of factors, while maintaining the third factor at a fixed central value, were constructed for both dependent variables.

#### 3.3. Optimization of the responses

The CD function was used to determine the best combinations of factor levels resulting in highest hydrolysis yields for both protein and carbohydrates. In this function, the desirability (*d*_*i*_) of each response is converted into a range from 0 to 1, when maximizing, *d*_*i*_ = 1 for high values and *d*_*i*_ = 0 for low values. The global desirability (*D*) is the geometric mean of the individual desirabilities (Eq 2).

$$D={\left(d_{1}∙d_{2}∙\ldots ∙d_{m}\right)}^{1/m}$$ (2)

where m is the number of response variables [35].

At the present work, CD was calculated under two different assumptions, either giving an equivalent desirability for the optimization of the results of hydrolysis for both protein and carbohydrates or a higher relevance to the first one.

## Results

### 1. Preliminary selection of the factors

Results of protein hydrolysis obtained from the initial factorial design are shown in Table 2. Values ranged from 35% to 73% of hydrolysis of protein (Table 2) and the statistical analysis indicated only a significant effect of those factors considered in the intestinal digestion, but no of those in the stomach hydrolysis (Table 3). Hence, these latter were not taken into account as independent variables in the next experiment, but were fixed at intermediate values within the assayed range (Table 3).

**Table 3:** Results from the factorial regression for the Plackett-Burman Design.

Variable	Range	Coefficient	SE	*p*–Value
Stom. pH	3.5–6.5	– 0.410	0.405	0.350
Int. pH	6.5–8.5	1.675	0.405	**0.006**
Stom. E:S	125–500 U mg^−1^ protein	– 0.121	0.405	0.776
Int. E:S	50–200 U mg^−1^ protein	1.269	0.405	**0.020**
Stom. T	4–8 h	– 0.318	0.405	0.462
Int. T	6–8 h	1.121	0.405	**0.033**
Lack of fit				0.219
R^2^	0.8678			
R^2^ (adjusted)	0.7135			
R^2^ (predicted)	0.2423			

Regression coefficients, R^2^, and Lack of fit for the dependent variable (aa). Significant regression coefficients are highlighted in bold.

### 2. Construction of the predictive models

Total amount of AA and RS released after the assays performed following the Central Composite Design (3 factors, 2 replicates, 3 base blocks, 6 total blocks, 40 total runs) are shown in Table 4. Values of protein hydrolysis ranged from 32% to 86%, while that of carbohydrates ranged from 10% to 36%. The values of the coefficients for those factors that showed a significant effect on the response variables are resumed in Table 5, expressed in coded units. The models showed a better fit for AA than for RS (adjusted R^2^ of 0.90 and 0.78, respectively), although both of them were significant, since the Lack-of-fit was *p* > 0.05 in both cases (0.65 and 0.16, respectively). A significant effect of the three factors considered on the hydrolysis of protein was evidenced, being more relevant the pH and the E:S ratio than the time of reaction. In addition, the increase in the values of any of the factors was directly correlated to the increase in the released AA. In addition, a significant interaction between pH and E:S ratio was noticed. In contrast, only the pH and E:S ratio showed a significant effect on the hydrolysis of carbohydrates, being the increase of pH negatively correlated to the amount of the released RS. The presence of a significant effect of E:S ratio in its quadratic form for the hydrolysis of both substrates indicated that the response was not simply linear within the range of the evaluated values.

**Table 4:** Central Composite Design with the intestinal parameters and the values obtained for the response variables (AA and RS).

Run order	Blocks	pH	E:S	T	AA Total amino acids released (mg)	% AA	RS Total reducing sugar released (mg)	% RS
1	6	7.5	125	5	131	45	24	22
2	6	7.5	247	7	182	63	16	14
3	6	7.5	3	7	100	35	40	36
4	6	5.9	125	7	106	37	34	31
5	6	7.5	125	7	146	50	15	14
6	6	9.1	125	7	215	74	17	16
7	6	7.5	125	7	146	50	17	15
8	6	7.5	125	9	156	54	14	12
9	2	8.5	50	6	131	45	23	21
10	2	7.5	125	7	177	61	17	16
11	2	7.5	125	7	173	60	17	16
12	2	6.5	50	8	105	36	24	22
13	2	8.5	200	8	249	86	14	13
14	2	6.5	200	6	133	46	21	19
15	4	8.5	200	6	186	64	14	13
16	4	6.5	50	6	107	37	29	26
17	4	7.5	125	7	159	55	18	16
18	4	8.5	50	8	150	52	18	16
19	4	7.5	125	7	147	51	16	15
20	4	6.5	200	8	151	52	21	19
21	1	7.5	125	7	159	55	19	17
22	1	8.5	50	8	132	46	21	19
23	1	8.5	200	6	209	72	16	14
24	1	6.5	50	6	92	32	38	35
25	1	7.5	125	7	160	55	16	15
26	1	6.5	200	8	140	48	20	18
27	5	6.5	200	6	131	45	16	15
28	5	8.5	50	6	130	45	26	24
29	5	6.5	50	8	122	42	30	27
30	5	7.5	125	7	157	54	19	17
31	5	7.5	125	7	171	59	17	15
32	5	8.5	200	8	233	80	14	12
33	3	7.5	125	5	134	46	16	14
34	3	5.9	125	7	119	41	31	29
35	3	7.5	125	9	179	62	14	12
36	3	7.5	247	7	191	66	15	13
37	3	7.5	125	7	179	62	17	16
38	3	7.5	3	7	111	38	28	26
39	3	9.1	125	7	196	68	11	10
40	3	7.5	125	7	142	49	10	10

**Table 5:** Models obtained from the Central Composite Design.

	% AA			% RS		
MODEL PARAMETERS	Coef.	SE	*p*-Value	Coef.	SE	*p*-Value
**Constant**	54.12	0.79	**0.000**	50,560	15,88	**0.003**
***Linear***						
**pH**	9.62	0.74	**0.000**	-3.88	0.58	**0.000**
**E:S**	9.38	0.74	**0.000**	61,768	19,260	**0.003**
**T**	3.57	0.74	**0.000**	-	-	-
***Squared***						
**pH^2^**	-	-	**-**	2.19	0.59	**0.001**
**E:S^2^**	-1.72	0.74	**0.027**	18,870	5,838	**0.001**
**T^2^**	-	-	-	-	-	-
***2-Way Interaction***						
pH * E:S	4.42	0.96	**0.000**	-	-	-
pH * T	-	-	-	-	-	-
E:S * T	-	-	**-**	-	-	-
**Lack-of-fit**			0.652			0.160
**R^2^**	0.93				0.83	
**R^2^ (adjusted)**	0.90				0.78	
**R^2^ (predicted)**	0.86				0.68	

Regression coefficients, R^2^, and Lack-of-fit test for the two dependent variables; % AA: protein digestibility (g/100 g substrate); % RS carbohydrate digestibility (g/100 g substrate). Significant regression coefficients are highlighted in bold. Hyphens indicate the non-significant terms that have been eliminated.

The mathematical models, showing coefficients for the significant factors in uncoded units were:

$$AA\;(g/100\;g\;substrate)=-8.2+2.25\;pH-0.241\;E:S+3.573\;T-0.000307\;{E:S}^{2}+0.0590\;{pH}^{*}E:S$$ (3)

$$RS\;(g/100\;g\;substrate)=182.5-36.75\;pH-15.09\;E:S+2.191\;{pH}^{2}+3.35\;{E:S}^{2}$$ (4)

**Fig 2:**
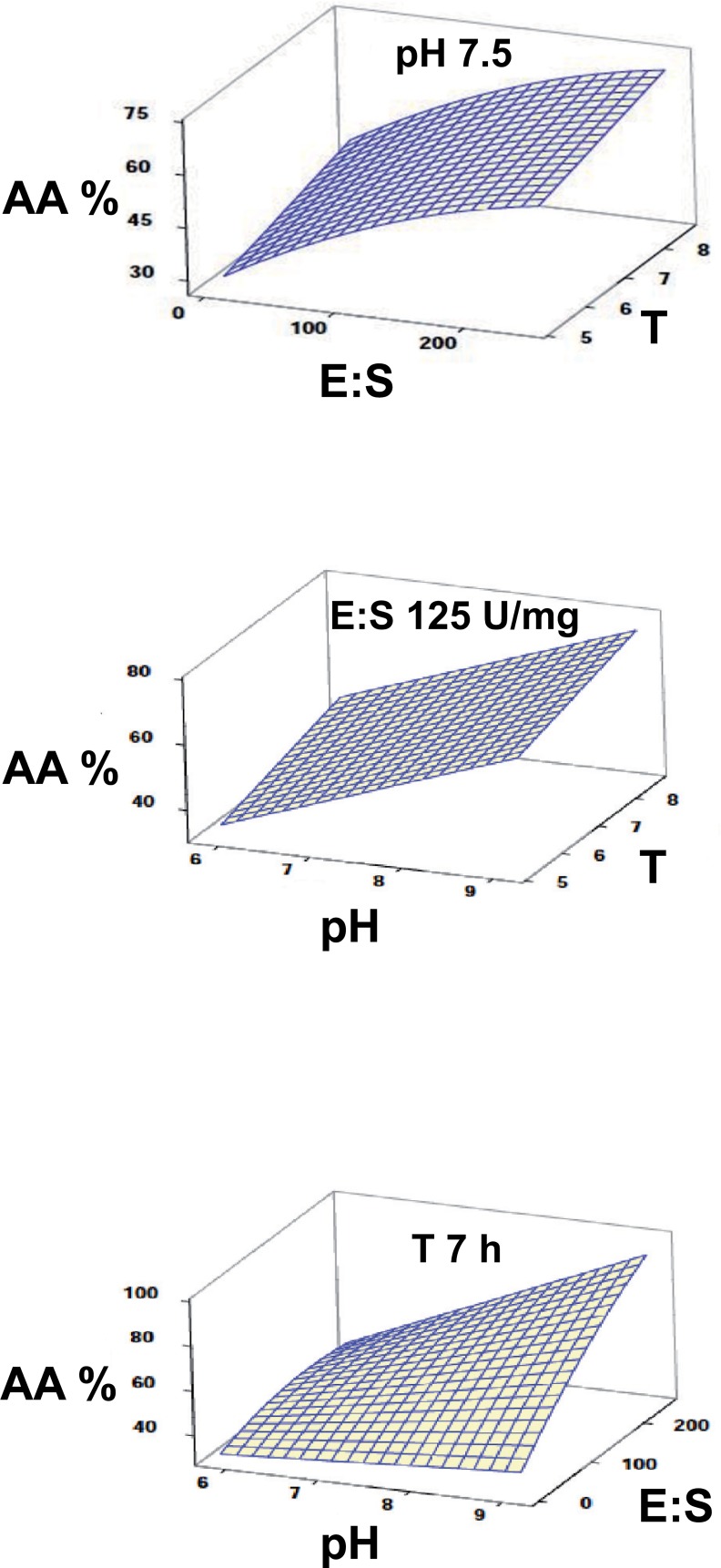
Surface plots describing the combined effect of the selected factors on protein hydrolysis in the gilthead seabream GIT.

**Fig 3:**
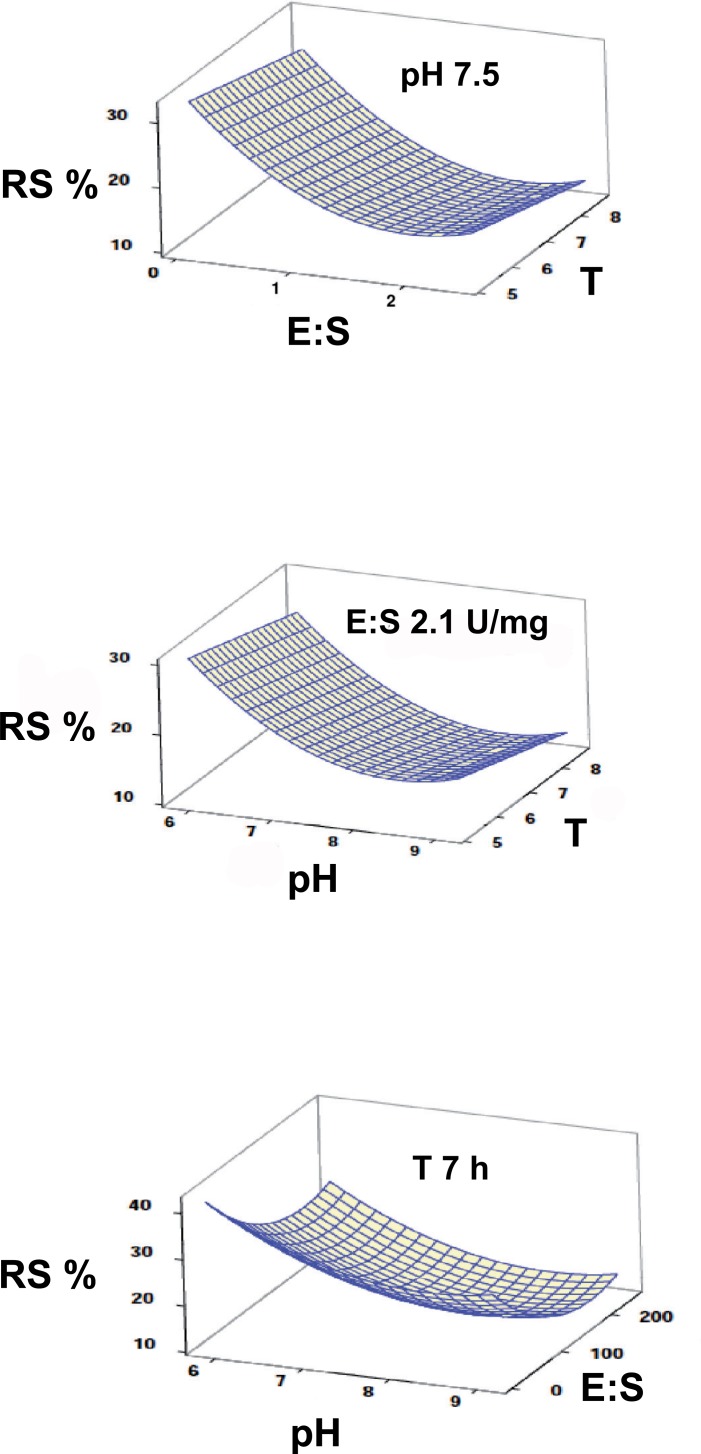
Surface plots describing the combined effect of the selected factors on carbohydrate hydrolysis in the gilthead seabream GIT.

The surface plots resuming the changes in the response variables as a function of each pair of factors evidenced great differences in the effect of the considered factors on the hydrolysis of either protein or carbohydrates (Figs 2 and 3). This pointed to a difficult compromise when trying to optimize both response variables at the same time.

### 3. Simultaneous Optimization of the responses–Composite Desirability function

**Fig 4:**
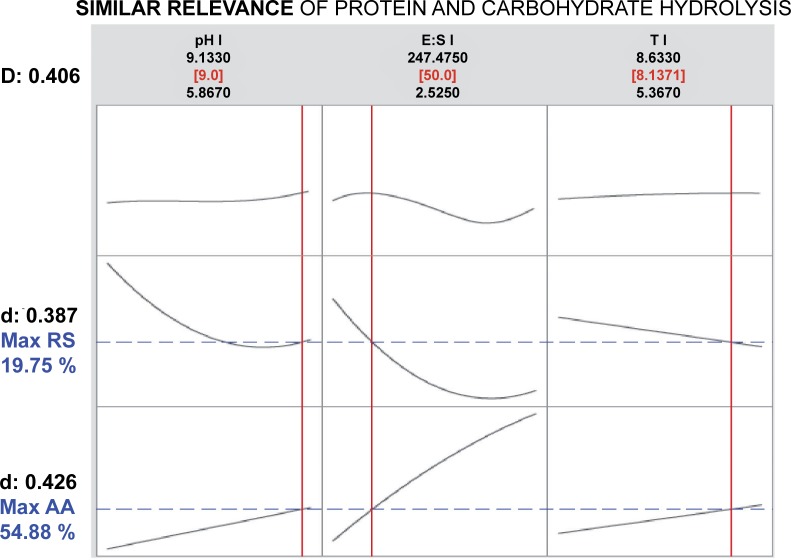
Optimization plot for AA and RS with the assumption of similar relevance of protein and carbohydrate hydrolysis. Solution (in red), predicted responses (in blue), simple and composite desirabilities are shown.

**Fig 5:**
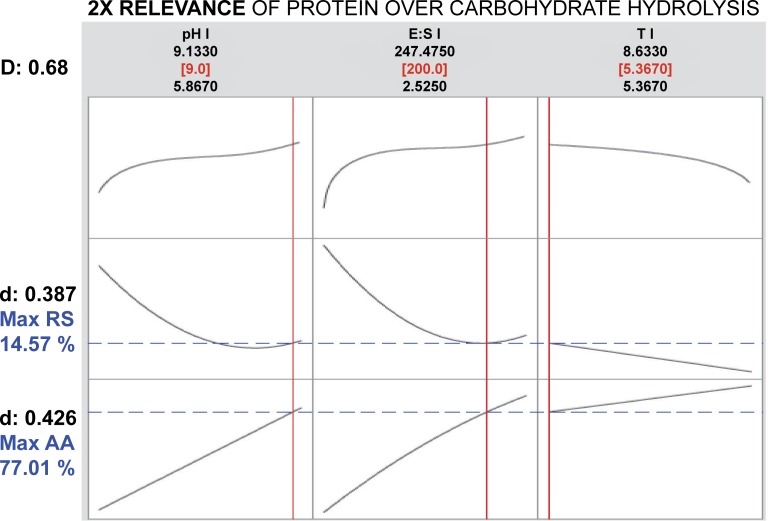
Optimization plot for AA and RS with the assumption of 2x relevance of protein over carbohydrate hydrolysis. Solution (in red), predicted responses (in blue), simple and composite desirabilities are shown.

Results obtained when using the CD to evaluate the possibility of a simultaneous optimization of protein and carbohydrate bioaccessibility in the gilthead seabream GIT are presented in Figs 4 and 5. In the first case, assuming a similar relevance for the hydrolysis of both substrates, the solution presented by the model shows that the selected values for each factor, within the assayed ranges, should be: a high pH (9.0), a medium E:S ratio (50 protease U mg^-1^ protein, 0.43 amylase U mg^-1^ carbohydrates), and a long reaction time (more than 8 h). Under such combination of factors, the hydrolysis of protein and carbohydrates should reach to 55% and 20%, respectively. The composed desirability reached a relatively low value of 0.41. Nevertheless, results may be different if maximization of protein hydrolysis is favoured over that of carbohydrates (i.e. giving its desirability a double weight than that of carbohydrates). In that case, the model showed an optimum response that maintained the same pH value (9.0), but increasing the E:S ratio (200 protease U mg^-1^ protein, 2 amylase U mg^-1^ carbohydrates) and reducing the reaction time to its lower value within the assayed range (about 5 h). Such combination of factors should result in a significant increase in the hydrolysis of protein (77%) and a slight reduction in that of carbohydrates (15%). The composed desirability in this case was improved, reaching a value of 0.68.

## Discussion

Although the factorial approach is routinely used to optimize conditions for enzyme hydrolysis in chemical reactors [36–38], to date only very few studies have adapted this type of experimental designs to *in vitro* assays simulating a biological digestion. Hollebeeck et al. (2103) [39] evaluated the influence of three continuous factors, in the range of values found in literature (pH, incubation time, and enzyme concentrations), on the simulated human digestion of standard macronutrients (starch, albumin, triolein) and validated the optimal conditions for salivary, gastric and duodenal steps of the digestion. A similar approach was carried out by Gilannejad et al. (2017) [17], who adapted this methodology to the conditions existing in the digestive tract of the flatfish *Solea senegalensis*, in order to assess the effect of the same factors on the intestinal hydrolysis of protein by this species. The present work also uses a combination of factorial design and *in vitro* assays to develop mathematical models for the enzyme hydrolysis that takes place in the gut of a fish. In this case, the models comprise a two-step hydrolysis (stomach and intestine), and the final objective was to assess which combination of factors determines the maximum bioavailability of two main nutrients, protein and carbohydrates, in the gut of the selected species. Since the hydrolysis of the two substrates is affected by the same combination of factors and takes place simultaneously during digestion, the net efficiency of the process was considered a good example of multiobjective optimization. Results obtained with the mathematical models were used to obtain conclusions that can be applicable to the processes that take place in the living fish.

As stated in the Introduction, a key point when using *in vitro* assays, as a tool to explain biological processes for a given species, is a previous collection of data to provide a robust physiological basis. As detailed by Moyano et al. (2014) [14], a great number of *in vitro* assays adapted to aquatic species fails, to different extent, at this point. As an example, some studies are carried out using commercial enzymes purified from microorganisms or from mammals. Although, different authors have demonstrated that fish digestive enzymes, particularly proteases, have different characteristics than those in other vertebrates; mainly their affinity for substrates, reaction speed, thermal optimum, and sensitivity to inhibitors [40–42]. Additionally, in most studies, there is a lack of explanation for the rationale to determine the amount of enzymes used in the assays, and it seems that they were selected only to produce a clearly measurable effect. The absence of a standardized relationship between the enzyme/substrate ratios used in the assays, which presumably is present in the digestive tracts of different species, may produce results that are not directly related to the *in vivo* processes. However, this factor has been strictly considered as an important point in *in vitro* digestibility assays conducted for terrestrial animals and humans [43, 44]. Finally, the pH of the *in vitro* assays should also be based on *in vivo* measurements, instead of the standardised use of pH 2.0 for acid phase. Real values are usually higher in the stomach or such a low value is achieved only for a short time in most of the examined fish species [22, 31, 45]. Similarly, the majority of the *in vitro* models in fish only simulate the alkaline step of digestion [14]. However, in species with carnivorous preference and a well-developed stomach, as in the case of gilthead seabream, *in vitro* methods involving both gastric and intestinal phases of digestion lead to more reliable hydrolysis models [15, 46]. Stomach digestion initiates the process of the breakage of the ingested food, and the partially hydrolysed proteins, as the result of the broad range of pepsin activity, is prepared for further hydrolysis in the intestine [15, 47, 48]. Besides, the gastric digestion, with its acidic pH, not only modulates the solubility of the substrates and the pepsin activity, but also plays an important role in inactivation of the protease inhibitors and increasing the minerals bioavailability [20, 27, 30]. All these points were carefully addressed in the present work, in order to establish the range of the factor considered in our experimental design as well as the *in vitro* assay procedures.

The mathematical optimization followed the steps detailed in the flow chart (Fig 1). The use of a preliminary factorial design to elucidate which factors may be significant or not when assayed within the physiological ranges, simplifies the DOE used in the RSM. In the present work, this initial screening evidenced a non-significant effect of all the considered factors simulating the stomach hydrolysis of the protein. This was somewhat surprising since the role of stomach in protein digestion of gilthead seabream is well established [22, 33]. Nevertheless, this could be explained considering that, according to the preliminary Plackett-Burman Design, many of the assays were performed at pH 6.5 (the highest limit defined for the stomach pH range). Under such conditions, pepsin cannot be activated and therefore, the net effect on the protein hydrolysis is negligible. This also points to the first practical conclusion of the model; a limited relevance of the stomach hydrolysis of proteins in the context of total protein hydrolysis taking place in the gut of the seabream. In other words, despite of the indispensable role of acid digestion in this species, the alkaline digestion plays a predominant role in the complete protein breakdown, possibly due to the limited stomach acidification capacity that suppresses the efficiency of the pepsin activity and/or the shorter food transit time in the stomach in comparison to the intestine. These findings cast doubt on the use of feed acidifiers for this species, due to a possible decrease in the intestinal pH that may impair the maximum hydrolysis and consequently the use of proteins.

In spite of having used the same range of values for the factors affecting the hydrolysis of both proteins and carbohydrates by the digestive enzymes of gilthead seabream, the mathematical models obtained using RSM for the release of either AA or RS were substantially different. While an increase in pH, E:S ratio, or reaction time resulted in a higher release of AA within the assayed range, the response obtained for RS was just the opposite and the higher values were obtained at the lower values of E:S or pH. Hence, the hydrolysis performed by amylase present in the enzyme extract seemed to be negatively affected by increasing pH, while reaction time did not exert a significant effect. This result was also highly surprising, considering that optimal pH of gilthead seabream amylase lies within the range of 6–8 used in the present assay [49]. Possible explanations for this response could be: i) the activity of amylase was affected by the presence of proteases in the enzyme extract or the peptides in the hydrolysis mixture; ii) the amylase function or determination of RS was interfered by the protein substrate or products of hydrolysis increasingly present in the reaction mixture; iii) the range of the factors selected favoured the activity of proteases but not that of amylase. Although the amylase inhibitory effect of some proteins or peptides has been previously described [50], this first hypothesis was rejected after testing that the activity of amylase was not only maintained but even slightly increased in enzyme extracts after long incubation times (see S1 Fig). On the other hand, a possible interference of free amino acids released by proteolysis on the quantification of reducing sugars when using DNSA has been reported by Sposina et al. (2102) [51], although this effect was mainly an overestimation instead of the observed decrease in our case. Additionally, an interactional effect of starch with haemoglobin [52] and iron ions [53], a by-product of haemoglobin hydrolysis, has been previously addressed. Therefore, another possibility is that these three-dimensional structures may prevent the amylase access to the starch molecules and its breakdown. According to the third hypothesis, it could be suggested that the conditions used in the assays (based on those measured in the live fish) seems to be more favourable for the hydrolysis of proteins than for carbohydrates. This is consistent with the biological response observed in carnivorous fish in relation to their limited ability to use carbohydrates either at digestive or metabolic level [54, 55].

As previously indicated, many biological processes are developed simultaneously, and, from the modelling perspective, a more realistic alternative is to take more than one criterion into account, an approach that may be closer to the way in which the nature has acted in the evolutionary process of optimization [56]. In this way, multi-criteria optimization plays an important role, since it considers the simultaneous optimization of several objectives. Multi-objective optimization has already been used in different biological contexts such as supervised and unsupervised classification of biological data, gene regulatory networks inference, sequence and structure alignment, protein structure prediction, and optimization of biochemical processes, among others [57]. One of the main objectives of the present work was to assess to which extent the simultaneous optimization of the mathematical models derived from the RSM for the hydrolysis of both substrates results in a response with a biological meaning. In this sense, the use of the CD of the functions used to explain the hydrolysis of either protein or carbohydrates offered interesting results. The values of combined AA and RS hydrolysis were close to those usually observed in the live fish, where the optimization of protein hydrolysis is favoured against that of carbohydrates. This suggests that a similar phenomenon can occur in the living fish and that the obtained model can be used to explain biological responses based on the results obtained from hydrolysis of digestive enzymes. From an applied point of view, this type of assay can be adapted to determine the optimal proportions of ingredients to be used in a feed to maximize the digestive release of both AA and RS. This may result in an increased bioavailability of these main nutrients and hence in better growth and feed efficiency.

To summarize, models resulting from RSM + *in vitro* assays may provide valuable information on the optimal combination of factors resulting in a higher bioaccessibilty of dietary substrates. Such models present a series of requirements in order to be adequately developed:

- A preliminary *in vivo* assessment of the range of values of factors under study.
- A careful choice of the substrates for each one of the nutrients and taking into account the possible interactions or interferences.
- High number of assays (replicates) to obtain a reasonably fitted model
- Validation of the model using *in vivo* responses obtained from similar factorial designs. Determination of the net bioaccessibility of the two assayed substrates must be carried out just after the hydrolysis in the proximal/medium intestine of the fish, but prior to the transformation in the distal portion caused by the microbial biomass.

When adequately developed, such models may be highly useful to predict the results from digestion of major nutrients when changing food/feeding parameters such as feeding frequency (modifies the digestion time), buffering capacity of the diet (modifies gastric and intestinal pH), and ration sizes (affect the E:S ratio).

## Supporting information

S1 Fig Maintenance of the activities of amylase and total alkaline protease in the intestinal extract of gilthead seabream over time. The extract was maintained at pH 7.5 (100 mM Tris-maleate buffer) and 25°C under continuous agitation during 4 hours. Values not sharing a common letter are significantly different with p < 0.05 (One-way ANOVA—Tukey's Multiple Comparison Test).
(DOCX)

## Abbreviations

AA: amino acids
CD: Composite Desirability
DNSA: 3,5-di-nitrosalicylic acid
DOE: Design of Experiments
E:S: Enzyme:substrate
GIT: Gastro intestinal tract
MWCO: Molecular Weight Cut-Off
RS: reducing sugars
RSM: Response Surface Methodology
T: Reaction time
U: Unit of enzyme activity
