# Supplementary material for: Modelling digestive hydrolysis of nutrients in fish using factorial designs and desirability function
Source: PLoS One. 2018 Nov 1;13(11):e0206556. doi: 10.1371/journal.pone.0206556 (PMC6211707; doi:10.1371/journal.pone.0206556)
Supplement: S1 Fig — The extract was maintained at pH 7.5 (100 mM Tris-maleate buffer) and 25°C under continuous agitation during 4 hours. Values not sharing a common letter are significantly different with p < 0.05 (One-way ANOVA—Tukey's Multiple Comparison Test). (DOCX) [file pone.0206556.s001.docx]

**S1 Fig. Maintenance of the activities of amylase and total alkaline protease in the intestinal extract of gilthead seabream over time.** The extract was maintained at pH 7.5 (100 mM Tris-maleate buffer) and 25 ºC under continuous agitation during 4 hours.

Values not sharing a common letter are significantly different with *p* < 0.05 (One-way ANOVA - Tukey's Multiple Comparison Test).
